# Herbal medicines in Brazil: pharmacokinetic profile and potential herb-drug interactions

**DOI:** 10.3389/fphar.2014.00162

**Published:** 2014-07-09

**Authors:** Andre L. D. A. Mazzari, Jose M. Prieto

**Affiliations:** Department of Pharmaceutical and Biological Chemistry, UCL School of PharmacyLondon, UK

**Keywords:** herb-drug interactions, cytochrome P450, glutathione, glucuronidation, P-glycoprotein, polymorphism, Brazil, pharmacovigilance

## Abstract

A plethora of active compounds found in herbal medicines can serve as substrate for enzymes involved in the metabolism of xenobiotics. When a medicinal plant is co-administered with a conventional drug and little or no information is known about the pharmacokinetics of the plant metabolites, there is an increased risk of potential herb-drug interactions. Moreover, genetic polymorphisms in a population may act to predispose individuals to adverse reactions. The use of herbal medicines is rapidly increasing in many countries, particularly Brazil where the vast biodiversity is a potential source of new and more affordable treatments for numerous conditions. Accordingly, the Brazilian Unified Public Health System (SUS) produced a list of 71 plant species of interest, which could be made available to the population in the near future. Physicians at SUS prescribe a number of essential drugs and should herbal medicines be added to this system the chance of herb-drug interactions further increases. A review of the effects of these medicinal plants on Phase 1 and Phase 2 metabolic mechanisms and the transporter P-glycoprotein was conducted. The results have shown that approximately half of these medicinal plants lack any pharmacokinetic data. Moreover, most of the studies carried out are *in vitro*. Only a few reports on herb-drug interactions with essential drugs prescribed by SUS were found, suggesting that very little attention is being given to the safety of herbal medicines. Here we have taken this information to discuss the potential interactions between herbal medicines and essential drugs prescribed to Brazilian patients whilst taking into account the most common polymorphisms present in the Brazilian population. A number of theoretical interactions are pinpointed but more pharmacokinetic studies and pharmacovigilance data are needed to ascertain their clinical significance.

## Introduction

Consumption of herbal medicines has been increasing worldwide over the past few years. In developed countries, such as the United Kingdom, around 50% of the population have used herbal medicines at least once in their life and, surprisingly, almost 100% of HIV patients in the country have admitted to using herbal medicines (Caminal Homar, 2005). In developing countries, the World Health Organization (WHO) estimates that 65–80% of the population relies on herbal medicines as a primary source of treatment (Rahman and Singhal, 2002). These statistics are in line with Brazil, where 66% of the population have no access to commercial medicines (Trojan-Rodrigues et al., 2012). Even when access is granted, popular use of herbal medicines is often due to poor medical and pharmaceutical assistance and the high cost of treatment with conventional drugs (Silveira et al., 2008). Brazilians are also becoming more interested in “safe” and “natural” treatments aimed to promote healthier living. A consequence of this increased use of phytotherapy has been a higher number of case reports on adverse reactions caused by uncontrolled consumption of herbal medicines (Silveira et al., 2008).

The Brazilian Health Surveillance Agency (ANVISA) is in charge of the regulation of herbal medicines in the country and since its creation in 1999, many advances have been made in order to control and ensure their efficacy and safety (Balbino and Dias, 2010). To guarantee that the phytomedicinal candidate is safe for consumption by humans, the Agency established a requirement that at least 20 years of prior traditional use must be attested. In the absence of this evidence the efficacy and safety of the candidate needs to be demonstrated by a point based system according to literature data, preclinical and clinical tests or indication that the herb is already included in the List of Simplified Registration of Herbal Medicines (ANVISA, 2010). Although these regulations are relevant to improve the safety and efficacy of herbal medicines, pharmacokinetic studies on the plants are not yet a regulatory requirement. As a consequence, there is a scarcity of this kind of data which is regarded as an oversight in terms of safety (Ribeiro et al., 2005).

The SUS is one of the biggest public health systems in the world, responsible for approximately 140 million Brazilian citizens (Mendes, 2013). In order to establish which medicines should be provided by SUS, the Brazilian Health Ministry produced a list of essential drugs according to the International Classification of Diseases (ICD) and epidemiological studies conducted nationwide (Saúde, 2010; SUS, 2010). Several herbal medicines are already part of this list, such as *Cynara scolymus L*., *Schinus terebinthifolius*, and *Rhamnus purshiana*. However, due to the extensive number of plant species possessing pharmacological activity used in Brazil, the Health Ministry determined that more herbal medicines should be provided by the System to the population, as part of one of the aims of the National Policy of Integrative and Complementary Practices (PNPIC). Consequently, the list of medicinal plants of interest of SUS (RENISUS) was published in 2008 and, from this point on, efforts have been concentrated on elucidating the efficacy and safety of the 71 plant species present on the list (Saúde, 2009; SUS, 2009; cf. Feijó et al., 2012).

The pharmacokinetic profile of pharmaceutical drugs is essential to determine whether or not they will interact with other therapeutic interventions (Ionescu and Caira, 2005). Polymorphism studies are also relevant because they may impact the capacity to metabolize xenobiotics, thus leading to adverse drug reactions (ADRs) within certain ethnicities (Suarez-Kurtz, 2005). However, pharmacokinetic studies on medicinal plants are very difficult to carry out because of their chemical complexity (Simões and Mariot, 2003). As a result, there is very little data for numerous native and exotic plants that are traditionally used in Brazil (He et al., 2010). The *in vitro* pharmacokinetic profile of a few herbal medicines can be found in the literature and, although these data are important, they are seldom used for the prediction of potential herb-drug interactions. Thus, the aim of this article is to provide an overview and critical evaluation of the pharmacokinetic data of medicinal plants to be used in the Brazilian health system. By discussing the potential herb-drug interactions with essential drugs upon Phase 1 and Phase 2 metabolic mechanisms and P-Glycoprotein activity, we intend to prompt race awareness on the safety of herbal medicines in Brazil.

## Methodology

A literature search was conducted using the Library of Medicine's PubMed database between May and June 2013. Included studies consisted of the reported effects of the medicinal plants of RENISUS list on the main liver metabolic enzymes, which are involved in Phase 1 (functionalization reactions mediated by cytochrome P450) and Phase 2 (glutathione conjugation, glucuronidation, sulfation, methylation and acetylation) metabolism and also the assessment of their effects on P-glycoprotein activity. The following combinations of keywords were used: “Plant name AND, 1A2, 3A4, 3A5, 3A7, 2C9, 2C19, 2D6, 2E1,” “Plant name AND glutathione,” or “Plant name AND gsh,” “Plant name AND glucuronidation” or “Plant name AND ugt,” “Plant name AND sulfation,” “Plant name AND sulfate conjugation,” “Plant name AND sulfotransferase,” “Plant name AND methylation,” “Plant name AND methyltransferase,” “Plant name AND acetylation,” “Plant name AND n-acetyltransferase,” “Plant name AND P-glycoprotein” or “Plant name AND Pgp.” EndNote web was the citation tool used to manage and organize all the references collected.

It is important to note that the list of Brazilian medicinal plants of interest of SUS encompass native and exotic adapted plant species and that we are aware of the chemical variability of the materials, which eventually may be harmonized by pharmacopoeial monographs of their respective countries.

## Phase 1 metabolism and the human liver cytochromes P450

Xenobiotic metabolism is normally divided into two phases: Phase 1 (functionalization reactions) and Phase 2 (conjugative reactions). Phase 1 reactions prepare the drug for Phase 2 metabolism by adding polar functional groups to the xenobiotic (Ionescu and Caira, 2005).

**Figure 1:**
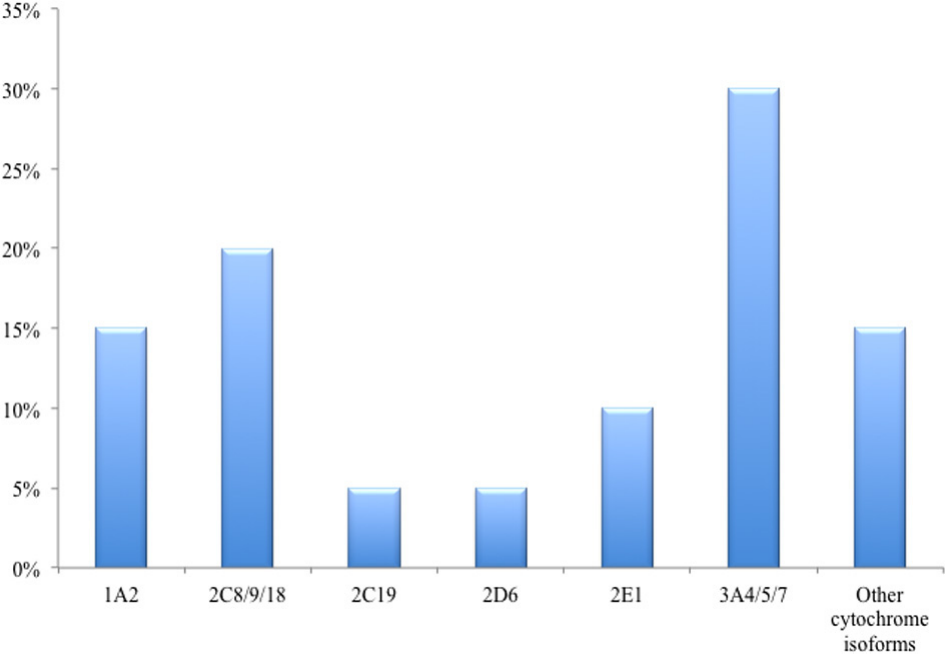
**The main liver cytochrome P450 isoenzymes and approximate percentage of expression**.

Human drug-metabolizing enzymes are present ubiquitously in the body. Over 50 human cytochromes P450 have already been isolated; the major ones found in the liver include CYP1A2, CYP2E1, CYP2C9/19, CYP2D6, and CYP3A4/5/7 (Figure 1) (Gibson and Skett, 2001). The CYP 1, 2, and 3 are the most abundant families of CYP metabolizing enzymes and the CYP1A2, CYP2C, and CYP3A4 isoforms account for about 30% of the metabolism of the majority of drugs (Atkinson, 2012).

The cytochrome P450 monooxygenase enzymes are located in the smooth endoplasmic reticulum of the liver and other extra hepatic tissues. Numerous drugs are metabolized by cytochrome P450 enzymes through oxidation reactions such as aromatic and aliphatic hydroxylation, epoxidation, *N*-dealkylation, *O*-dealkilation, *S*-dealkylation, oxidative deamination, *N*-oxidation, *S*-oxidation, phosphothionate oxidation, dehalogenation, and alcohol oxidation. Reduction, hydrolysis and hydration are other examples of Phase 1 reactions catalyzed by the cytochrome P450 (Ionescu and Caira, 2005).

Similar to conventional drugs, herbal medicines also undergo Phase 1 and Phase 2 metabolism in order to be excreted from the body. If a herbal medicine is concomitantly used with a conventional drug, for example, the first may inhibit or induce the activity and expression of a specific cytochrome that could be the same one responsible for the metabolism of the latter, leading to herb-drug interactions (Hu et al., 2005).

### Genetic polymorphism

Responses to drug consumption differ among individuals due to the variability of CYP content. A predefined dosage of a medicine might be enough to exert a pharmacological effect in one patient but it may be necessary to be adjusted for another patient to achieve the same effect. This can be explained by genetic polymorphisms within CYPs that can affect the metabolism of xenobiotics in general, leading to changes in drug response and increased risk of ADRs (Zhou et al., 2009). For example, the biodisponibility of Omeprazole, which is a drug that is metabolized by CYP3A4 and CYP 2C19, demonstrated to be slower in Mexicans when compared with Caucasians but faster than that of Asians. The ethnic mixture of the Mexican population is a plausible explanation for differences in metabolic response compared to other ethnic groups (Gonzalez et al., 2003).

The genomic diversity of the Brazilian population is a result of the genetic admixture of three groups: Europeans, Africans and Amerindians. Due to this very distinctive miscegenation, polymorphisms in cytochromes levels are undoubtedly present among Brazilians (Suarez-Kurtz, 2005). The Brazilian National Pharmacogenetics/Pharmacogenomics Network (REFARGEN) carried out studies in four regions of Brazil in order to map the genetic diversity of the population (Suarez-Kurtz, 2004). The study divided the population into three distinct races: white, brown and black. The results obtained from REFARGEN showed that numerous polymorphisms were found on cytochromes 2C9, 2C19, 2D6, and 3A5.

Taking all these points into consideration, from the data gathered in our literature review we will summarize some potentially clinically relevant pharmacokinetic effects of herbal medicines in metabolic enzymes responsible for Phase 1 and Phase 2 metabolisms and P-glycoprotein activity. Also, the presence of polymorphisms among the Brazilian population and its implication in the metabolism were assessed. Some interactions between essential drugs and herbal medicines in Brazil found in the literature will be shown, which will eventually need to be put to test in the laboratory or followed up epidemiologically.

### Effects of herbal medicines in phase 1 enzymes and herb-drug interactions

#### CYP1A2

This cytochrome isoform is mainly found in the liver (15%) and it metabolizes almost 20% of the current therapeutic arsenal (Wang and Zhou, 2009). Amid the 71 plant species, nine of them were found to interfere with the activity of CYP1A2 (Table 1). Examples of essential drugs, which are substrates for the CYP1A2, include the highly popular painkiller Acetaminophen, the beta-blocker Propranolol, the antidepressant Clomipramine and the anticoagulant Warfarin.

**Table 1:** **Medicinal plant species listed in RENISUS with reported effects of on CYP1A2**.

**Plant species/Family**	**Effects on CYP1A2**	**References**
*Allium sativum* (Aliaceae)	+	Le Bon et al., 2003
*Curcuma longa* (Zingiberaceae)	+	Thapliyal et al., 2002
*Eucalyptus globulus* (Myrtaceae)	−	Unger and Frank, 2004
*Glycine max* (Leguminosae)	−	Shon and Nam, 2004
*Harpagophytum procumbens* (Pedaliaceae)	NE, −	Unger and Frank, 2004; Modarai et al., 2011
*Mentha piperita* (Lamiaceae)	−	Unger and Frank, 2004
*Phyllanthus amarus* (Euphorciaceae)	−	Hari Kumar and Kuttan, 2006
*Punica granatum* (Lythraceae)	−	Faria et al., 2007a
*Trifolium pratense* (Fabaceae)	−	Unger and Frank, 2004

*+, Enzyme induction; −, Enzyme inhibition; NE, No Effect*.

Acetaminophen (Paracetamol) is bioactivated via CYP1A2, CYP3A4, and CYP2E1, resulting in the production of the toxic compound N-acetyl–*p*–benzoquinone (NAPQI). In normal conditions, the drug is detoxified by conjugation with glutathione. However, in cases of overdose, this metabolic route becomes saturated, increasing the bioactivation of NAPQI and its binding to other molecules, such as proteins, resulting in damages to the liver due to cell death (Lee et al., 2001). Intake of Acetaminophen with *Allium sativum* and *Curcuma longa* could theoretically increase the toxicity of the drug due to accumulation of NAPQI caused by induction of CYP1A2. On the other hand, consumption of Acetaminophen with medicinal plants such as *Phyllantus amarus, Mormodica charantia, Eucalyptus globulus, Glycine max, Harpagophytum procumbens, Mentha piperita, Trifolium pratense* and *Punica granatum* based remedies may decrease levels of this toxic metabolite because of CYP1A2 enzyme inhibition.

Although it appears that garlic consumption can put the integrity of hepatocytes at risk, a study conducted by Gwilt et al. (1994) proved that the garlic extract administration has little effect on the metabolism of Acetaminophen. In this study 16 male nonsmokers took 10 ml of garlic extract daily for 3 months. Acetaminophen was administered at five different time points: before garlic treatment, at the end of the first, second, and third month of garlic extract consumption and 1 month after interruption of garlic treatment. The results demonstrated that garlic extract does not interfere with the oxidative pathway of Acetaminophen and therefore it does not represent a potential risk for hepatocytes.

The beta-blocker Propranolol and the tricyclic antidepressant Clomipramine are essential drugs extensively prescribed in Brazil. The biotransformation of Propranolol and Clomipramine starts with the *N*-dealkilation, governed by *S*-mephenytoin (CYP1A2) for the former and the *N*-demethylation of the side chain of the molecule of the latter to form the active metabolite desmethylclomipramine (DCIP) (Nielsen et al., 1996). Therefore, plant species that inhibit CYP1A2 must be avoided during the treatment period with these drugs. This is, however, theoretical and in this case no reports have so far been found in the literature.

The anticoagulant Warfarin is indicated for the treatment of venous thrombosis and pulmonary embolism. The pharmacokinetics of this drug can easily be altered by CYP1A2 inhibitors. A rare case of interaction of warfarin with garlic supplements has been reported, leading to excessive bleeding (Baxter and Stockley, 2008). Other cases of herb-drug interactions were detected after co-administration of *Curcuma longa, Glycine max, Harpagophytum procumbens, Punica granatum*, and *Trifolium pratense* with Warfarin, resulting in pharmacokinetic alterations (Heck et al., 2000; Cambria-Kiely, 2002; Ramsay et al., 2005; Komperda, 2009; Liu et al., 2013).

CYP1A2 is considered to be highly inducible and polymorphic. Two polymorphisms were found to be common in South Brazilians: the CYP1A2^*^1F and ^*^1K alleles. They cause higher and decreased enzyme expression, respectively (Kohlrausch et al., 2014). Their high frequency in Southern Brazilians suggests that chances of ADRs may be increased within this group.

#### CYP2C9

The CYP2C9 is the major CYP2C isoform found in the human liver and typical substrates are molecules that contain an anionic site and a hydrophobic site (Mo et al., 2009). This CYP enzyme metabolizes approximately 15% of clinical drugs, including the nonsteroidal anti-inflammatory (NSAID) Ibuprofen, the antihypertensive Losartan, the antidepressant Fluoxetine, the antiepileptic Phenytoin and the anti-hypercholesterolemic Fluvastatin (Baxter and Stockley, 2008; Berka et al., 2011). Certain endogenous bioactive substances such as steroids, melatonin, retinoids and arachidonic acid are also metabolized by this CYP family (Mo et al., 2009).

Ibuprofen is a common medication prescribed by SUS to combat pain and inflammation. The drug is also included in the WHO Model List of Essential Medicines as a drug for pain and palliative care and as an antimigraine medicine (Medicines, 2012; WHO, 2012, 2013). As mentioned above, Ibuprofen is mostly metabolized by CYP2C9 leading to the formation of the active metabolite (*S*)-ibuprofen. Herbal species that are substrates for CYP2C9 (Table 2), may inhibit the formation of (*S*)-ibuprofen, potentially leading to therapeutic failure (Mo et al., 2009).

**Table 2:** **Medicinal plant species listed in RENISUS with reported effects of on CYP2C9**.

**Plant species/Family**	**Effects on CYP2C9**	**References**
*Allium sativum* (Aliaceae)	−, +	Foster et al., 2001; Ho et al., 2010
*Eucalyptus globulus* (Myrtaceae)	−	Unger and Frank, 2004
*Harpagophytum procumbens* (Pedaliaceae)	NE, −	Modarai et al., 2011
*Mentha piperita* (Lamiaceae)	−	Unger and Frank, 2004
*Punica granatum* (Lythraceae)	−	Hanley et al., 2012
*Trifolium pratense* (Fabaceae)	−	Unger and Frank, 2004
*Zingiber officinale* (Zingiberaceae)	−	Kimura et al., 2010

*+, Enzyme induction; −, Enzyme inhibition; NE, No Effect*.

The active metabolites E-3174, norfluoetine, 4′-HPPH and 6-hydroxy fluvastatin, are formed through the action of CYP2C9 on the essential drugs Losartan, Fluoxetine, Phenytoin and Fluvastatine, respectively (von Moltke et al., 1997; Scripture and Pieper, 2001; Joy et al., 2009). Concomitant consumption of those drugs along with any of the plant species listed on Table 2 could impact on the formation of these substances.

The CYP2C9^*^2 and ^*^11 alleles were found in higher frequency in white Brazilians, whereas CYP2C9*3 was mostly found in browns and CYP2C9^*^5 in the black population. These polymorphisms decrease the enzyme activity *in vitro* (Zhou et al., 2009) therefore consumption of medicinal plants such as garlic, eucalyptus, devil's claw, mentha, pomegranate, red clover and ginger may further inhibit their function or expression, potentially causing herb-drug interactions in these ethnic groups.

#### CYP2C19

Cytochrome P450 2C19 is not only involved in the metabolism of a range of drugs but it also plays a crucial role in the detoxification and inactivation of some potential carcinogens (Wang et al., 2013). CYP2C19 is responsible for the metabolism of about 10% of prescribed drugs, including a number of essential ones such as the proton pump inhibitor Omeprazole, the tricyclic antidepressant Amitriptyline, the selective serotonin reuptake inhibitor Fluoxetine, the benzodiazepine Diazepam and the barbiturate Phenobarbital (Zhou et al., 2009). About 24 variants of CYP2C19 are known of which three of them were found by REFARGEN in the Brazilian population: CYP2C19^*^2, CYP2C19^*^3, and CYP2C19^*^17. The alleles ^*^2 and ^*^3 appear not to effect the enzyme activity, whereas CYP2C19^*^17 increases *in vitro* activity and is the most prevalent.

The plant species *Allium sativum*, *Eucalyptus globulus*, *Mentha piperita*, and *Trifolium pratense* were found to be CYP2C19 inhibitors (Table 3). Due to the extensive use of these herbal medicines that are metabolized by CYP2C19, herb-drug interactions at this level could be very frequent but so far no clinical report has been found.

**Table 3:** **Medicinal plant species listed in RENISUS with reported effects of on CYP2C19**.

**Plant species/Family**	**Effects on CYP2C19**	**References**
*Allium sativum* (Aliaceae)	−	Foster et al., 2001
*Eucalyptus globulus* (Myrtaceae)	−	Unger and Frank, 2004
*Harpagophytum procumbens* (Pedaliaceae)	NE	Modarai et al., 2011
*Mentha piperita* (Lamiaceae)	−	Unger and Frank, 2004
*Trifolium pratense* (Fabaceae)	−	Unger and Frank, 2004

*+, Enzyme induction; −, Enzyme inhibition; NE, No Effect*.

#### CYP2D6

Although the level of expression of the cytochrome P450 2D6 in the human liver is only about 5%, it metabolizes about 25% of all medications in human liver (Ionescu and Caira, 2005). Essential drugs such as the beta-blockers Propafenone and Timolol, the antidepressant Amitriptyline, the antipsychotic Haloperidol and Risperidone, and the antihistamine Chlorphenamine are metabolized by this isoform (Zhou et al., 2009). Polymorphisms on CYP2D6 are the most studied among the CYPs. To date, 72 variants of this isoform were found in humans and 16 were detected in Brazilians according to REGARGEN: CYP2D6^*^1, ^*^2, ^*^3, ^*^4, ^*^5, ^*^9, ^*^10, ^*^17, ^*^29, ^*^34, ^*^35, ^*^39, ^*^41, ^*^1XN, ^*^2XN, and ^*^4XN. An increase in enzyme activity was found on the alleles ^*^1XN and ^*^2XN, whereas a decrease of activity was found on ^*^9, ^*^10, ^*^17, ^*^29, and ^*^41.

The popular medicinal plants *Eucalyptus globulus, Harpagophytum procumbens, Mentha piperita, Phylanthus amarus, Punica granatum*, and *Trifolium pratense* are shown to inhibit the activity of CYP2D6 in liver cells (Table 4). Brazilians who take these herbal drugs may have increased chances of ADRs, although no clinical reports were found in the literature.

**Table 4:** **Medicinal plant species listed in RENISUS with reported effects of on CYP2D6**.

**Plant species/Family**	**Effects on CYP2D6**	**References**
*Allium sativum* (Aliaceae)	NE	Markowitz et al., 2003
*Eucalyptus globulus* (Myrtaceae)	−	Unger and Frank, 2004
*Harpagophytum procumbens* (Pedaliaceae)	NE, −	Modarai et al., 2011
*Mentha piperita* (Lamiaceae)	−	Unger and Frank, 2004
Phyllanthus amarus (Euphorbiaceae)	−	Hari Kumar and Kuttan, 2006
*Punica granatum* (Lythraceae)	−	Usia et al., 2006
*Trifolium pratense* (Fabaceae)	−	Unger and Frank, 2004

*+, Enzyme induction; −, Enzyme inhibition; NE, No Effect*.

#### CYP2E1

The cytochrome P450 2E1 represents 10% of the total CYPs expressed in the human liver and it is well known for its involvement in the metabolism of ethanol to acetaldehyde, and accordingly it is rapidly induced after ethanol ingestion (Anzenbacher and Anzenbacherova, 2001). This cytochrome isoform is responsible for the activation of some carcinogens, procarcinogens and toxicants and it metabolizes mainly low-molecular-weight compounds. CYP2E1 also has the ability to produce reactive intermediates, leading to the formation of free radicals such as superoxide, hydroxyl radical, and lipid peroxides (Neafsey et al., 2009). Polymorphisms on CYP2E1 in the Brazilian population have not yet been mapped by REFARGEN, but one case of herb-drug interaction involving this enzyme has been reported (Hau et al., 2009). According to our literature search, *Allium sativum, Momordica chrantia, Phyllanthus amarus, Phyllanthus urinaria*, and *Punica granatum* decrease levels and activity of CYP2E1 in the liver (Table 5). As Acetaminophen is also metabolized by CYP2E1, NAPQI can be also formed at this metabolic route. Therefore, consumption of these medicinal plants could reduce the formation of the toxic metabolite. For example, species of the *Phyllanthus* genus are traditionally used for conditions such as jaundice, gonorrhea, frequent menstruation, diabetes and as a pain killer (Naaz et al., 2007; Patel et al., 2011). A study revealed that *Phyllanthus urinaria* inhibits CYP2E1 activity in hepatocytes and it also attenuates acetaminophen induced hepatotoxicity in mice. The experiment was conducted by treating a total of 37 mice with acetaminophen at a dose of 550 mg/kg of body weight on day one in order to induce liver injury. The mice were then divided into two groups: the first group was treated with *Phyllanthus urinaria* extract from day 2 to 4 whereas the second group just received water. The final results indicated that the herbal drug was able to inhibit the formation of NAPQI and, consequently, prevent liver failure (Hau et al., 2009).

**Table 5:** **Medicinal plant species listed in RENISUS with reported effects of on CYP2E1**.

**Plant species/Family**	**Effects on CYP2E1**	**References**
*Allium sativum* (Aliaceae)	−	Le Bon et al., 2003
*Curcuma longa* (Zingiberaceae)	NE	Salama et al., 2013
*Glycine max* (Leguminosae)	NE	Shon and Nam, 2004
Momordica charantia (Cucurbitaceae)		Raza et al., 1996
Phyllanthus amarus (Euphorbiaceae)		Hari Kumar and Kuttan, 2006
Phyllanthus urinaria (Euphorbiaceae)		Shen et al., 2008
*Punica granatum* (Lythraceae)		Faria et al., 2007a

*+, Enzyme induction; −, Enzyme inhibition; NE, No Effect*.

#### CYP3A

The most abundant subfamily of cytochromes is CYP3A (it represents about 30% of the entire CYP450 enzymes in the liver) and it is responsible for processing more than 50% of therapeutic drugs. CYP3A exists in the body in three isoforms: 3A4, 3A5, and 3A7. CYP3A5 is more often detected in adolescents than in adults, where it is hardly inducible. CYP3A4 is mostly glucocorticoid-inducible and CYP3A7 (found only in fetal livers) has a role in hydroxylations of allylic and benzylic carbon atoms (Ionescu and Caira, 2005). According to our findings, practically all the medicinal plants that demonstrated activity in the Phase 1 metabolism are substrates for the CYP3A family (Table 6). Macrolide antibiotics, anti-arrythmics, benzodiazepines, immune modulators, HIV antivirals, antihistamines, calcium channel blockers and HMG CoA reductase inhibitors are examples of classes of medications metabolized by the CYP3A subfamily (Zhou et al., 2009). The probability of herb-drug interactions with this isoform is high and therefore a particular attention to all CYP3A substrates should be given in order to avoid herb-drug interactions.

**Table 6:** **Medicinal plant species listed in RENISUS with reported effects of on CYP3A**.

**Plant species/Family**	**Effects on CYP3A**	**References**
*Allium sativum* (Aliaceae)	NE, −(^*^/^**^/^***^)	Foster et al., 2001; Hajda et al., 2010
*Chamomilla recutita* (Asteraceae)	−(^*^)	Budzinski et al., 2000
*Curcuma longa* (Zingiberaceae)	NE(^*^)	Graber-Maier et al., 2010
*Eucalyptus globulus* (Myrtaceae)	−(^*^)	Unger and Frank, 2004
*Foeniculum vulgare* (Apiaceae)	−(^*^)	Subehan et al., 2006; Subehan Zaidi et al., 2007
*Harpagophytum procumbens* (Pedaliaceae)	NE, -(^*^)	Unger and Frank, 2004; Modarai et al., 2011
*Mentha piperita* (Lamiaceae)	−(^*^)	Unger and Frank, 2004
*Mormodica charantia* (Cucurbitaceae)	−(^*^)	Raza et al., 1996
*Phyllanthus amarus* (Euphorbiaceae)	−(^*^/^**^/^***^)	Hari Kumar and Kuttan, 2006
*Punica granatum* (Lythraceae)	−(^*^/^**^/^***^)	Faria et al., 2007a
*Trifolium pratense* (Fabaceae)	−(^*^)	Budzinski et al., 2000
*Uncaria tomentosa* (Rubiaceae)	−(^*^)	Budzinski et al., 2000
*Zingiber officinale* (Zingiberaceae)	(^*^)	Kimura et al., 2010

* CYP3A4,

** CYP3A5,

*** CYP3A7/+,

*Enzyme induction; −, Enzyme inhibition; NE, No Effect*.

HIV positive patients are commonly treated with the essential drug Saquinavir. At the same time, dietary supplements such as garlic and/or immune system boosters like Cat's claw can be used to help prevent, combat and improve health. In 1998, a case report was published demonstrating that Saquinavir is a substrate of CYP3A4 and that garlic (an inhibitor of CYP3A) was able to interfere with the metabolism of the drug, leading to failures in therapy and possible drug resistance (Chen et al., 2011). Another case report showed that a 45-year-old woman, who was HIV positive and had cirrhosis caused by a hepatitis C infection was not responding satisfactorily to the treatment of the anti-HIV drugs. The patient had no good adherence to the HIV treatment so was then asked whether she was making use of any other medicines, including herbal medicines. Surprisingly, the patient was taking *Uncaria tomentosa* preparation for 2 months, probably to enhance the immune system. She was asked to cease taking of the herbal medicine and after 15 days the C_min_ values of the anti-HIV drugs were normalized. As shown in Table 6, *Uncaria tomentosa* has a high inhibitory capability to CYP3A4 causing an increase in the C_min_ values for antiretroviral agents leading to an increased risk of toxicity. The combined therapy with these two agents has been shown to be a potential risk for HIV patients (Lopez Galera et al., 2008).

Another example of a CYP3A substrate is the HMG CoA reductase inhibitor Atorvastatin. This essential medicine is indicated for patients with dyslipidemia, reducing levels of total cholesterol, low-density lipoprotein cholesterol (LDL), triglycerides, very low-density lipoprotein cholesterol (VLDL) and for increasing high-density lipoprotein cholesterol (HDL) levels. A pharmacokinetic study conducted in 2012 showed that the half-life of the drug was increased in rats treated with *Allium sativum* due to inhibition of CYP3A4 by the herbal medicine (Reddy et al., 2012).

REFARGEN has reported CYP3A5 polymorphisms among Brazilians with the most frequent alleles being CYP3A5^*^1, ^*^3, ^*^6, and ^*^7. The alleles ^*^3 and ^*^6 decrease enzyme activity *in vitro*, whereas ^*^1 and ^*^7 showed no effects (Zhou et al., 2009). Interestingly, the allele ^*^3 is the most common variation of this isoform in Brazil. Because it reduces the enzyme activity, intake of herbal medicines that exert the same effect should be strongly avoided in order to prevent herb-drug interactions.

## Phase 2 metabolism and potential pharmacokinetic herb-drug interactions

Phase 2 metabolism reactions (or Conjugation reactions) occur when metabolic enzymes react with functional groups of a drug that was formed during the Phase 1 process. Endogenous species, such as a sugar or an amino acid, are added to the drug in order to increase the polarity to allow its elimination. The two main Phase 2 biotransformation reactions are glutathione conjugation and glucuronidation but the other conjugative reactions such as sulfonation, methylation and acetylation are also relevant (Atkinson, 2012).

### Glutathione conjugation (GSH)

Glutathione is a tripeptide present in high concentrations in the liver. It has a protective role removing toxic electrophilic compounds from the body (Ionescu and Caira, 2005). Conjugation with glutathione avoids the reaction of electrophilic compounds to nucleophilic ones in macromolecules such as proteins and nucleic acids. When the conjugate is formed, it has to undergo further metabolic reactions in order to form mercapturic acid. The final product is then eliminated from the organism (Sies and Ketterer, 1988).

The plant species on Table 7 can affect the glutathione levels in liver cells according to our literature search. The metabolite NAPQI, generated by the CYP isoforms 2E1, 3A4, and 1A2, undergoes glutathione conjugation (Baxter and Stockley, 2008). When production of NAPQI exceeds liver stores of glutathione, the organ is damaged due to the attachment of NAPQI to liver proteins (Alipour et al., 2013). Thus, a combined therapy of acetaminophen and herbal species that deplete glutathione levels, listed in Table 7, should be monitored.

**Table 7:** **Medicinal plant species listed in RENISUS with reported effects of on glutathione levels**.

**Plant species/Family**	**Effects on glutathione levels**	**References**
*Achillea millefoilum* (Asteraceae)	+	Potrich et al., 2010
*Allium sativum* (Aliaceae)	+	Ip and Lisk, 1997
*Aloe vera/Aloe barbadensis* (Aloaceae)	−, +	Kaithwas et al., 2011; Hegazy et al., 2012
*Anacardium occidentale* (Anacardiaceae)	+	Singh et al., 2004
*Baccharis trimera* (Asteraceae)	−	Nogueira et al., 2011
*Bauhinia forficata* (Caesalpiniaceae)	−	Damasceno et al., 2004
*Bauhinia variegata* (Caesalpiniaceae)	+	Rajkapoor et al., 2006
*Calendula officinalis* (Asteraceae)	+	Preethi and Kuttan, 2009
*Chamomilla recutita* (Asteraceae)	+	Al-Hashem, 2010
*Croton cajucara* (Euphorbiaceae)	+	Rabelo et al., 2010
*Curcuma longa* (Zingiberaceae)	+	Rong et al., 2012
*Cynara scolymus* (Asteraceae)	+, NE	Miccadei et al., 2008
*Foeniculum vulgare* (Apiaceae)	+	Zhang et al., 2012
*Glycine max* (Leguminosae)	+	Barbosa et al., 2011
*Mentha pulegium* (Lamiaceae)	+	Alpsoy et al., 2011
*Mentha piperita* (Lamiaceae)	+	Sharma et al., 2007
*Mikania glomerata* (Asteraceae)	NE	Barbosa et al., 2012
*Momordica charantia* (Cucurbitaceae)	+	Raza et al., 2000, 1996
*Phyllanthus amarus* (Euphorbiaceae)	+	Kumar and Kuttan, 2004, 2005; Karuna et al., 2009; Maity et al., 2013
*Phyllanthus niruri* (Euphorbiaceae)	+	Bhattacharjee and Sil, 2006; Manjrekar et al., 2008
*Psidium guajava* (Myrtaceae)	+	Tandon et al., 2012
*Punica granatum* (Myrtaceae)	+, −	Faria et al., 2007b; Dassprakash et al., 2012
*Ruta graveolens* (Rutaceae)	+	Ratheesh et al., 2011
*Zingiber officinale* (Zingiberaceae)	+, NE	Ajith et al., 2007

*+, Enzyme induction; − Enzyme inhibition; NE, No Effect*.

A study demonstrated that consumption of garlic extract protects hepatocytes against acetaminophen-induced glutathione depletion. In order to measure glutathione levels, hepatocytes were isolated from male Sprague-Dawley rats and incubated with different concentrations of garlic extract at three different times: before, at the same time and 30 min after addition of acetaminophen. The results were collected at different time points (from 0 to 150 min) and it was observed that the intake of garlic extract is able to protect hepatocytes against acetaminophen-induced toxicity by increasing intracellular GSH levels (Anoush et al., 2009).

Studies on polymorphisms in the Brazilian population of enzymes in glutathione conjugation, such as glutathione *S*-tranferase (GST) have not been carried out by REFARGEN. However, some reports revealed its existence among this population. A study conducted by Rossini et al. (2002) reported the existence of GST polymorphisms in a group of 519 Brazilians from Rio de Janeiro. GSTM1 is involved in the detoxification of polycyclic aromatic hydrocarbons and some mutagens, whereas GSTT1 catalyzes the metabolism of halomethanes by human erythrocytes. It was found that the null allele, i.e., no expression of the enzyme, was detected in approximately 10% of the studied population. Null individuals are generally more susceptible to DNA damage by the action of the compounds already mentioned. Although we cannot extrapolate this data for all Brazilians, the existence of these polymorphisms is thought to be quite common among multi-ethnic populations. Therefore, the extensive list of plant species found to affect the glutathione conjugation mechanism plus the presence of such polymorphisms could potentially increase chances of herb-drug interactions.

### Glucuronidation

Glucuronidation is a mechanism in which a glucuronide is formed by the reaction between the electrophilic C-1 atom of the pyranose acid ring of the co-factor UDPGA (uridine 5′-diphosphate-glucuronic acid) with the substrate catalyzed by UDP-glucuronosyltransferases (UGTs). Uridine diphosphate glucuronosyltransferases (UGT's) are the most important Phase 2 enzymes and they are found in the highest amount among all conjugation enzymes in the liver (Ionescu and Caira, 2005; Caira and Ionescu, 2006). This is the most important form of conjugation of xenobiotics with chemical groups such as alcohols, phenols, hydroxylamines, carboxylic acids, amines, sulphonamides, and thiols (Gibson and Skett, 2001).

Phase 1 metabolites of nonsteroidal anti-inflammatory drugs (NSAIDs), such as Ibuprofen, predominantly undergo glucuronidation in order to be eliminated from the organism (Kuehl et al., 2005). *Allium sativum* increases the expression of UGTs whereas *Curcuma longa* inhibits the expression of these enzymes in the liver (Table 8). Therefore, the pharmacokinetic of this class of drugs can be compromised by consumption of any of these herbal species.

**Table 8:** **Medicinal plant species listed in RENISUS with reported effects of on UGT levels**.

**Plant species/Family**	**Effects on UGT levels**	**References**
*Allium sativum* (Aliaceae)	+	Ip and Lisk, 1997
*Curcuma longa* (Zingiberaceae)	−	Naganuma et al., 2006

*+, Enzyme induction; −, Enzyme inhibition; NE, No Effect*.

REFARGEN has not yet published data about polymorphisms on UDP- glucuronosyltransferase enzymes. However, information about polymorphic UGTs and its impact on ADRss and cancer susceptibility have been reported (Guillemette, 2003).

### Other conjugative reactions

Besides the two main liver conjugation reactions (glutathione conjugation and glucuronidation), the xenobiotic transformation could also happen by sulfation (or sulfate conjugation), methylation (or methyl conjugation), and acetylation (Ionescu and Caira, 2005).

Sulfation is another Phase 2 detoxification mechanism that is recognized to be the major conjugation pathway for phenols, alcohols, amines and thiols (Ionescu and Caira, 2005). Before the Phase 1 metabolites undergo sulfate conjugation, the inorganic sulfate has to be activated via ATP to form adenosine-5′-phosphosulphate (APS) and, consequently, 3′-phosphoadenosine-5′-phosphosulphate (PAPS). Sulfotransferase enzymes (SULT) will then catalyze the detoxification of essential drugs, such as Salbutamol and Acetaminophen by transferring a sulfuryl group from PAPS to an acceptor molecule (Gibson and Skett, 2001). Methylation is the mainly metabolic pathway for endogenous compounds but it could be also the route of many drugs and xenobiotics in general (Weinshilboum, 1988). Methyl conjugate reactions are only possible in the presence of the co-factor *S*-adenosylmethionine (SAM) and will result in the formation of *O*-methylated, *N*-methylated, and *S*-methylated products (Gibson and Skett, 2001). The liver is the primary site for acetylation reactions, but the reaction could also happen in some extra hepatic sites, such as the spleen, lungs and gut. To summarize, the *N*-acetyltransferases will catalize the transferring of the co-factor acetyl-coenzyme A (acetyl-CoA) to aromatic amines and sulphonamides and form the polar metabolites (Ionescu and Caira, 2005).

After the literature search, it was concluded that the 71 plant species that are the focus of this review need to have their pharmacokinetic profile studied, because no such data has been published. The risk of potential herb-drug interactions caused by herbal medicines that are metabolized through sulfation, methylation and acetylation pathways is therefore increased and it could be already affecting the efficacy of conventional drugs. REFARGEN has not published any data about genetic polymorphisms in the Brazilian population for these conjugation mechanisms to date.

## Effects of plant species on P-glycoprotein (Pgp) activity and herb-drug interaction

Drug-transporter proteins are known to allow xenobiotics to cross biological membranes, the most well-known one being Pgp. This protein plays a role as an efflux pump that pushes metabolites and drugs out of the cells which can result in pharmacokinetic alterations (Williamson et al., 2009). Some metabolites generated by the metabolism of herbal medicines can be pumped back to the lumen due to the activity of P-glycoprotein and hence, oral delivery can be compromised (Butterweck et al., 2004). Among the herbal medicines that are the object of this review, *Achillea millefolium* demonstrated inhibition of P-glycoprotein whereas *Allium sativum* activates the transporter (Table 9). Herb-drug interactions have already been reported on Pgp activity. For example, the HIV-protease inhibitor Saquinavir (substrate of CYP3A4) is absorbed in the intestine via P-glycoprotein. As garlic extracts may induce Pgp activity, the outcome of concomitant consumption of the herbal medicine with the essential drug could potentially reduce the bioavailability of the latter (Williamson et al., 2009).

**Table 9:** **Medicinal plant species listed in RENISUS with reported effects of on P-glycoprotein activity**.

**Plant species/Family**	**Effects on P-glycoprotein activity**	**References**
*Achillea millefolium* (Asteraceae)	−	Haidara et al., 2006
*Allium sativum* (Aliaceae)	+	Hajda et al., 2010
*Curcuma longa* (Zingiberaceae)	NE	Graber-Maier et al., 2010

*+, Efflux increased; −, Efflux decreased*.

Studies have shown that the gene ABCB1, which encodes Pgp, is very polymorphic and that the pharmacokinetics of several Pgp substrates could be significantly altered (Scheiner et al., 2010). REFARGEN has identified three ABCB1 polymorphisms in the Brazilian population: 1236T (rs1128503), 2677non G (rs2032582), and 3435T (rs1045642). Pharmacokinetic studies of drugs that affect the regulation of ABCB1, will help in the future in order to avoid herb-drug interactions on that level.

## Pharmacovigilance of herbal medicines

The increasing consumption of herbal medicines in the world raises a concern about their rational use by the population. The WHO has recognized the potential risks of uncontrolled use of herbal medicines in conjunction with other medicines and hence, this Organization issued in 2004 the “Guidelines on safety monitoring of herbal medicines in pharmacovigilance systems.” These guidelines indicate how member countries should include herbal medicines to an existent pharmacovigilance system to facilitate the exchange of information (WHO, 2004). After the establishment of ANVISA in 1999, efforts were concentrated on the creation of a national system of pharmacovigilance, and in 2001 the National Center for Drug Monitoring (CNMM) was founded. Notifications of ADRs in Brazil is currently made through an electronic system called NOTIVISA and these data come from three different sources: a “Sentinel Network,” which provides information on adverse reactions related to the use of health products in hospitals, such as drugs, blood and hemoderivatives; pharmacies will report suspected cases of drug reactions (including herb-drug interactions); and a scheme of “Spontaneous Notifications” that can be made by any health professional registered into the electronic system (Mendes et al., 2008).

The number of ADRs notified by pharmacovigilance systems in the world until 2012 resulting from herb-drug interactions was 811, with *Allium sativum, Mentha piperita, Zingiber officinale* and *Glycine max* among the top 20 of the most commonly reported herbal medicines. Brazil was not included in the list of countries which have reported herb-drug interactions, demonstrating the difficulties in collection of such data by the pharmacovigilance system (Skalli and Soulaymani Bencheikh, 2012). Since the creation of NOTIVISA in 2008, there are no notifications of adverse reactions to herbal medicines, demonstrating that the new system created more obstacles to the users (Balbino and Dias, 2010).

Most of the problems related to the notification of ADRs due to herbal medicine consumption occur mainly because Practitioners and other health professionals are not well trained to detect the origin of the reaction. Besides, patients in general do not inform their use of herbal medicines and Physicians may not have sufficient knowledge about the effects of the phytomedicines in the body. Thus, educational campaigns should be utlilized in order to emphasize the rational use of medicinal plants and also to encourage health professionals to notify any adverse effect that might be a result of herb-drug interaction (Balbino and Dias, 2010).

## Conclusions

In 2008 Brazil became a model country, clearly defining a positive list of herbal medicines considered “essential,” and implementing an agenda to make possible the complete implementation of herbal medicines at a clinical level in the foreseeable future. This is a leading example within the current global trend toward the integration of herbal medicines into the healthcare system.

Our review highlights that little is known about native Brazilian plants. Most of the data is related to herbal medicines used worldwide, such as garlic, mint and devil's claw. We tried to interpret this in the context of the genetic makeup of the Brazilian population and how this may interact with essential drugs prescribed within the Brazilian healthcare system. It is evident that an enormous task should be undertaken to understand the pharmacokinetics of most local plant species.

Ensuring safety of herbal medicines goes beyond the interpretation of the preclinical or clinical evidence. Quality is also on the basis of safety and this is a challenging task in the field of Phytotherapy, with many drugs lacking identifiable active principles. Healthcare professionals and the public alike have to be well trained/informed in the use of the final medicinal product. Moreover, only a robust system of pharmacovigilance will help in the identification of relevant safety issues at a population level. These are not necessarily mirroring the preclinical and clinical data available.

At this stage, the preclinical pharmacokinetic profile of medicinal plants can only be evaluated with available data in literature. Polymorphism studies in the Brazilian population have been a valuable source of information that can help with the assessment of ADRs and herb-drug interactions.

By tentatively exploring any potential interaction between herbal medicines and other essential medicines in the Brazilian system, we hope to open up future research to both bridge the gaps in knowledge and support future risk assessment of herb-drug combinations. Again, our results highlight that much more work is needed as over half of the medicinal plants lack data. Only after this is done can we start assessing the risk of herb-drug interactions when medicinal plants are taken with any essential drug.

Safety of herbal medicines finally lies in the hands of the patients. In this regard, healthcare professionals need proper training on how to advise the patient and the labeling of the product must anticipate further doubts when the patient cannot communicate with the professional.

### Conflict of interest statement

The authors declare that the research was conducted in the absence of any commercial or financial relationships that could be construed as a potential conflict of interest.
