# Corrigendum: Herbal medicines in Brazil: pharmacokinetic profile and potential herb-drug interactions

**DOI:** 10.3389/fphar.2015.00023

**Published:** 2015-03-10

**Authors:** Andre L. D. A. Mazzari, Jose M. Prieto

**Affiliations:** Pharmacognosy, School of Pharmacy, University College LondonLondon, UK

**Keywords:** herb-drug interactions, cytochrome P450, glutathione, glucuronidation, P-glycoprotein, polymorphism, Brazil, pharmacovigilance

We noticed that some symbols were lacking in Table 5 and some of the Plant Family names were not currently accepted.

We hereby present the Tables 1–9 with all symbols and botanical names re-checked.

**Table 1:** **Medicinal plant species listed in RENISUS with reported effects of on CYP1A2**.

**Plant species/Family**	**Effects on CYP1A2**	**References**
*Allium sativum* (Amaryllidaceae)	+	Le Bon et al., 2003
*Curcuma longa* (Zingiberaceae)	+	Thapliyal et al., 2002
*Eucalyptus globulus* (Myrtaceae)	–	Unger and Frank, 2004
*Glycine max* (Leguminosae)	–	Shon and Nam, 2004
*Harpagophytum procumbens* (Pedaliaceae)	NE, –	Unger and Frank, 2004; Modarai et al., 2011
*Mentha piperita* (Lamiaceae)	–	Unger and Frank, 2004
*Phyllanthus amarus* (Phyllanthaceae)	–	Hari Kumar and Kuttan, 2006
*Punica granatum* (Lythraceae)	–	Faria et al., 2007a
*Trifolium pratense* (Leguminosae)	–	Unger and Frank, 2004

*+, Enzyme induction; –, Enzyme inhibition; NE, No Effect*.

**Table 2:** **Medicinal plant species listed in RENISUS with reported effects of on CYP2C9**.

**Plant species/Family**	**Effects on CYP2C9**	**References**
*Allium sativum* (Amaryllidaceae)	–,+	Foster et al., 2001; Ho et al., 2010
*Eucalyptus globulus* (Myrtaceae)	–	Unger and Frank, 2004
*Harpagophytum procumbens* (Pedaliaceae)	NE, –	Modarai et al., 2011
*Mentha piperita* (Lamiaceae)	–	Unger and Frank, 2004
*Punica granatum* (Lythraceae)	–	Hanley et al., 2012
*Trifolium pratense* (Leguminosae)	–	Unger and Frank, 2004
*Zingiber officinale* (Zingiberaceae)	–	Kimura et al., 2010

*+, Enzyme induction; –, Enzyme inhibition; NE, No Effect*.

**Table 3:** **Medicinal plant species listed in RENISUS with reported effects of on CYP2C19**.

**Plant species/Family**	**Effects on CYP2C19**	**References**
*Allium sativum* (Amaryllidaceae)	–	Foster et al., 2001
*Eucalyptus globulus* (Myrtaceae)	–	Unger and Frank, 2004
*Harpagophytum procumbens* (Pedaliaceae)	NE	Modarai et al., 2011
*Mentha piperita* (Lamiaceae)	–	Unger and Frank, 2004
*Trifolium pratense* (Leguminosae)	–	Unger and Frank, 2004

*+, Enzyme induction; –, Enzyme inhibition; NE, No Effect*.

**Table 4:** **Medicinal plant species listed in RENISUS with reported effects of on CYP2D6**.

**Plant species/Family**	**Effects on CYP2D6**	**References**
*Allium sativum* (Amaryllidaceae)	NE	Markowitz et al., 2003
*Eucalyptus globulus* (Myrtaceae)	–	Unger and Frank, 2004
*Harpagophytum procumbens* (Pedaliaceae)	NE, –	Modarai et al., 2011
*Mentha piperita* (Lamiaceae)	–	Unger and Frank, 2004
*Phyllanthus amarus* (Phyllanthaceae)	–	Hari Kumar and Kuttan, 2006
*Punica granatum* (Lythraceae)	–	Usia et al., 2006
*Trifolium pratense* (Leguminosae)	–	Unger and Frank, 2004

*+, Enzyme induction; –, Enzyme inhibition; NE, No Effect*.

**Table 5:** **Medicinal plant species listed in RENISUS with reported effects of on CYP2E1**.

**Plant species/Family**	**Effects on CYP2E1**	**References**
*Allium sativum* (Amaryllidaceae)	–	Le Bon et al., 2003
*Curcuma longa* (Zingiberaceae)	NE	Salama et al., 2013
*Glycine max* (Leguminosae)	NE	Shon and Nam, 2004
*Momordica charantia* (Cucurbitaceae)	–	Raza et al., 1996
*Phyllanthus amarus* (Phyllanthaceae)	–	Hari Kumar and Kuttan, 2006
*Phyllanthus urinaria* (Phyllanthaceae)	–	Shen et al., 2008
*Punica granatum* (Lythraceae)	–	Faria et al., 2007a

*+, Enzyme induction; –, Enzyme inhibition; NE, No Effect*.

**Table 6:** **Medicinal plant species listed in RENISUS with reported effects of on CYP3A**.

**Plant species/Family**	**Effects on CYP3A**	**References**
*Allium sativum* (Amaryllidaceae)	NE, –(^*^/^**^/^***^)	Foster et al., 2001; Hajda et al., 2010
*Chamomilla recutita* (Compositae)	–(^*^)	Budzinski et al., 2000
*Curcuma longa* (Zingiberaceae)	NE(^*^)	Graber–Maier et al., 2010
*Eucalyptus globulus* (Myrtaceae)	–(^*^)	Unger and Frank, 2004
*Foeniculum vulgare* (Apiaceae)	–(^*^)	Subehan et al., 2006, 2007
*Harpagophytum procumbens* (Pedaliaceae)	NE, –(^*^)	Unger and Frank, 2004; Modarai et al., 2011
*Mentha piperita* (Lamiaceae)	–(^*^)	Unger and Frank, 2004
*Momordica charantia* (Cucurbitaceae)	–(^*^)	Raza et al., 1996
*Phyllanthus amarus* (Phyllanthaceae)	–(^*^/^**^/^***^)	Hari Kumar and Kuttan, 2006
*Punica granatum* (Lythraceae)	–(^*^/^**^/^***^)	Faria et al., 2007a
*Trifolium pratense* (Leguminosae)	–(^*^)	Budzinski et al., 2000
*Uncaria tomentosa* (Rubiaceae)	–(^*^)	Budzinski et al., 2000
*Zingiber officinale* (Zingiberaceae)	(^*^)	Kimura et al., 2010

* *CYP3A4*,

** *CYP3A5*,

*** *CYP3A7/+, Enzyme induction;–, Enzyme inhibition; NE, No Effect*.

**Table 7:** **Medicinal plant species listed in RENISUS with reported effects of on glutathione levels**.

**Plant species/Family**	**Effects on glutathione levels**	**References**
*Achillea millefoilum* (Compositae)	+	Potrich et al., 2010
*Allium sativum* (Amaryllidaceae)	+	Ip and Lisk, 1997
*Aloe vera/Aloe barbadensis* (Xanthorrhoeaceae)	–,+	Kaithwas et al., 2011; Hegazy et al., 2012
*Anacardium occidentale* (Anacardiaceae)	+	Singh et al., 2004
*Baccharis trimera* (Compositae)	–	Nogueira et al., 2011
*Bauhinia forficata* (Leguminosae)	–	Damasceno et al., 2004
*Bauhinia variegata* (Leguminosae)	+	Rajkapoor et al., 2006
*Calendula officinalis* (Compositae)	+	Preethi and Kuttan, 2009
*Chamomilla recutita* (Compositae)	+	Al-Hashem, 2010
*Croton cajucara* (Euphorbiaceae)	+	Rabelo et al., 2010
*Curcuma longa* (Zingiberaceae)	+	Rong et al., 2012
*Cynara scolymus* (Compositae)	+, NE	Miccadei et al., 2008
*Foeniculum vulgare* (Apiaceae)	+	Zhang et al., 2012
*Glycine max* (Leguminosae)	+	Barbosa et al., 2011
*Mentha pulegium* (Lamiaceae)	+	Alpsoy et al., 2011
*Mentha piperita* (Lamiaceae)	+	Sharma et al., 2007
*Mikania glomerata* (Asteraceae)	NE	Barbosa et al., 2012
*Momordica charantia* (Cucurbitaceae)	+	Raza et al., 1996; Raza et al., 2000
*Phyllanthus amarus* (Phyllanthaceae)	+	Kumar and Kuttan, 2004, 2005; Karuna et al., 2009; Maity et al., 2013
*Phyllanthus niruri* (Phyllanthaceae)	+	Bhattacharjee and Sil, 2006; Manjrekar et al., 2008
*Psidium guajava* (Myrtaceae)	+	Tandon et al., 2012
*Punica granatum* (Lythraceae)	+,–	Dassprakash et al., 2012; Faria et al., 2007b
*Ruta graveolens* (Rutaceae)	+	Ratheesh et al., 2011
*Zingiber officinale* (Zingiberaceae)	+, NE	Ajith et al., 2007

*+, Enzyme induction; –, Enzyme inhibition; NE, No Effect*.

**Table 8:** **Medicinal plant species listed in RENISUS with reported effects of on UGT levels**.

**Plant species/Family**	**Effects on UGT levels**	**References**
*Allium sativum* (Amaryllidaceae)	+	Ip and Lisk, 1997
*Curcuma longa* (Zingiberaceae)	–	Naganuma et al., 2006

*+, Enzyme induction;–, Enzyme inhibition; NE, No Effect*.

**Table 9:** **Medicinal plant species listed in RENISUS with reported effects of on P-glycoprotein activity**.

**Plant species/Family**	**Effects on P-glycoprotein activity**	**References**
*Achillea millefolium* (Compositae)	–	Haidara et al., 2006
*Allium sativum* (Amaryllidaceae)	+	Hajda et al., 2010
*Curcuma longa* (Zingiberaceae)	NE	Graber-Maier et al., 2010

*+, Efflux increase; –, Efflux decrease*.

Apologies for any inconvenience this may have caused.

## Conflict of interest statement

The authors declare that the research was conducted in the absence of any commercial or financial relationships that could be construed as a potential conflict of interest.

